# Cost-Effectiveness of Cranberries vs Antibiotics to Prevent Urinary Tract Infections in Premenopausal Women: A Randomized Clinical Trial

**DOI:** 10.1371/journal.pone.0091939

**Published:** 2014-04-04

**Authors:** Judith E. Bosmans, Mariëlle A. J. Beerepoot, Jan M. Prins, Gerben ter Riet, Suzanne E. Geerlings

**Affiliations:** 1 Department of Health Sciences and EMGO Institute for Health and Care Research, Faculty of Earth and Life Sciences, VU University Amsterdam, Amsterdam, The Netherlands; 2 Department of Internal Medicine, Division of Infectious Diseases, Academic Medical Center, Amsterdam, The Netherlands; 3 Department of General Practice, Academic Medical Center, Amsterdam, The Netherlands; Glaxo Smith Kline, Denmark

## Abstract

**Background:**

Urinary tract infections (UTIs) are common and result in an enormous economic burden. The increasing prevalence of antibiotic-resistant microorganisms has stimulated interest in non-antibiotic agents to prevent UTIs.

**Objective:**

To evaluate the cost-effectiveness of cranberry prophylaxis compared to antibiotic prophylaxis with trimethoprim-sulfamethoxazole (TMP-SMX) over a 12 month period in premenopausal women with recurrent UTIs.

**Materials and Methods:**

An economic evaluation was performed alongside a randomized trial. Primary outcome was the number of UTIs during 12 months. Secondary outcomes included satisfaction and quality of life. Healthcare utilization was measured using questionnaires. Missing data were imputed using multiple imputation. Bootstrapping was used to evaluate the cost-effectiveness of the treatments.

**Results:**

Cranberry prophylaxis was less effective than TMP-SMX prophylaxis, but the differences in clinical outcomes were not statistically significant. Costs after 12 months in the cranberry group were statistically significantly higher than in the TMP-SMX group (mean difference €249, 95% confidence interval 70 to 516). Cost-effectiveness planes and cost-effectiveness acceptability curves showed that cranberry prophylaxis to prevent UTIs is less effective and more expensive than (dominated by) TMP-SMX prophylaxis.

**Conclusion:**

In premenopausal women with recurrent UTIs, cranberry prophylaxis is not cost-effective compared to TMP-SMX prophylaxis. However, it was not possible to take into account costs attributed to increased antibiotic resistance within the framework of this randomized trial; modeling studies are recommended to investigate these costs. Moreover, although we based the dosage of cranberry extract on available evidence, this may not be the optimal dosage. Results may change when this optimal dosage is identified.

**Trial Registration:**

ISRCTN.org ISRCTN50717094

## Introduction

Urinary Tract Infections (UTIs) are very common, especially in women. Almost half of all women report at least one UTI sometime during their lifetime, and after an initial UTI, 20% to 30% of women experience a recurrence [1]. Although acute UTI is considered a benign condition, acute UTI can have negative consequences. Women find the main symptoms of UTI, painful and frequent micturition, bothersome and these symptoms have a negative impact on quality of life [2], [3], [4], [5]. The high incidence of UTI results in considerable costs due to physician visits, diagnostic tests and medication. It was estimated that total costs due to UTI amounted to $2.3 billion in the US in 2010 [6].

For premenopausal women with more than 2 UTIs per year, low-dose antibiotic prophylaxis is commonly recommended [7]. However, this may lead to drug resistance of both the causative microorganisms and the indigenous flora [8]. The increasing prevalence of isolates of *Escherichia coli* (the most prevalent uropathogen) that are resistant to antimicrobial agents has stimulated interest in novel non-antibiotic methods for the prevention of UTIs [9].

Cranberries have been used in the prevention of UTIs for many years. The mechanism of action has not been completely elucidated, but cranberries contain fructose and type A proanthocyanidins (PACs), which in urine can inhibit the adherence of type 1 and P fimbriae of *E coli* to the uroepithelial cell receptors [10], [11], [12]. A meta-analysis of the results of 4 well-conducted randomized controlled trials showed that, in women with recurrent UTIs, cranberry products in various forms and dosages as compared with placebo or other control interventions appeared to reduce the occurrence of symptomatic UTI, but not statistically significantly so (relative risk 0.74, 95% CI 0.42–1.31) [13]. Only one study evaluated the cost-effectiveness of prophylactic treatment with cranberry products in comparison with placebo and showed that cranberry products reduced the mean number of UTIs during a year, but at considerably increased costs [14]. Standard prophylaxis for recurrent UTIs consists of low-dose antibiotics (e.g., trimethoprim-sulfamethoxazole [TMP-SMX]). However, most trials compared cranberries with placebo or no intervention.

Therefore, the aim of this paper was to evaluate the cost-effectiveness of cranberry prophylaxis (capsules, 500 mg twice daily) compared to antibiotic prophylaxis with trimethoprim-sulfamethoxazole (TMP-SMX, 480 mg once daily) over a 12 month period in premenopausal women with recurrent UTIs.

## Materials and Methods

### Design

This economic evaluation was conducted alongside a randomized trial that was conducted from January 1, 2005 through August 31, 2007 in The Netherlands. The study protocol was approved by the medical ethics committees of all 10 participating centers, and all participants gave written informed consent before inclusion. Procedures were in accordance with the Helsinki Declaration, and this trial is registered in the International Standard Randomized Controlled Trial Number Register (ISRCTN 50717094). The methodological details of the trial are reported in detail elsewhere and briefly summarized here [15]. The protocol for this trial and supporting CONSORT checklist are available as supporting information; see Checklist S1 and Protocol S1.

### Patients

Premenopausal women, 18 years of age or older, reporting to have had at least 3 symptomatic UTIs in the year preceding enrollment were eligible. Patients were living in the community and were recruited through advertisement in women's magazines and in journals, through primary care physicians' referral, and from secondary or tertiary hospitals all over the Netherlands.

Exclusion criteria were symptoms of a UTI at inclusion, use of antibiotics or cranberries in the previous 2 weeks, contraindications for TMP-SMX (e.g., known allergy) or cranberries (oral anticoagulants [16] or renal stones [17]), relevant interactions of either treatment with existing medication, (desire for) pregnancy, breastfeeding, and a history of renal transplantation.

### Randomization

The coordinating center (Academic Medical Center, Amsterdam, The Netherlands) prepared drug randomization lists for each study site in advance. Masking of patients and investigators was achieved by double-dummy dosing. Concealed randomization was ensured by using computer-aided block randomization (block size was kept secret), with prestratification by center and presence (yes/no) of complicating host factors, defined as functional or structural abnormalities of the urinary tract, metabolic and/or hormonal abnormalities, or impaired host responses [18].

### Intervention

Women were randomized to 12 months' ingestion of either (1) 1 tablet with 480 mg TMP-SMX at night and 1 placebo capsule twice daily or (2) 1 capsule with 500 mg cranberry extract (Cran-Max; Proprietary Nutritionals, Inc, Kearny, New Jersey) twice daily and 1 placebo tablet at night. The amount of Type A PACs in the cranberry extract was 9.1 mg/g [15]. A twice-daily capsule with 500 mg cranberry extract was prescribed on the basis of the results of a previous laboratory study showing anti-adhesion activity in the urine within 2 hours and persisting for up to 10 hours following cranberry juice consumption [19].

### Outcome measures

Immediately before the start of the study medication and monthly thereafter, until 3 months after discontinuation of the study medication, the women received the same questionnaire addressing UTI symptoms, adverse events (AEs), infections other than UTIs, and antibiotic consumption.

The primary clinical outcome was the number of symptomatic UTIs (clinical recurrences [CRs]) over 12 months. A CR was defined as a UTI on the basis of a woman's subjective report of clinical symptoms, usually dysuria, frequency, and/or urgency. Secondary clinical outcomes included quality of life and satisfaction with the prophylactic treatment. Quality of life was measured using the SF-36 at baseline, 6 months and 12 months [20]. The SF-36 scores were used to estimate utilities according to the SF-6D tariff developed by Brazier et al. [21] Quality-Adjusted Life-Years (QALYs) were calculated by multiplying the utilities with the amount of time a patient spent in a particular health state. Transitions between health states were linearly interpolated. Satisfaction with the prophylactic treatment received was measured at 12 months of follow-up. Satisfaction was measured on a visual analogue scale ranging from 0 (not satisfied) to 100 (very satisfied).

UTI-related healthcare utilization in the past month and absenteeism due to UTI in the past week was assessed monthly using questionnaires. Direct healthcare costs included visits to the general practitioner, visits to the outpatient clinic, the number of tests performed (ultrasound, X-ray, laboratory tests, etcetera), hospital admissions, visits to other primary healthcare providers (physiotherapist, complementary therapist), visits to an emergency department, and home care. Indirect non-healthcare costs were also measured and included informal care, paid help, and additional costs due to UTI (bed linen, underwear). Indirect costs included absenteeism from paid work. Resource utilization was valued using Dutch standard costs [22]. All costs were adjusted to the year 2009 using consumer price indices if necessary [23]. Discounting was not necessary, because follow-up was limited to 12 months. Table 1 lists the cost categories and prices used in this economic evaluation.

**Table 1:** Cost categories and prices (€, 2009) used in this economic evaluation.

Category	Price (€, 2009)
*Direct healthcare costs*	
General practitioner, visit	28.00
General practitioner, visit outside office hours	75.50
General practice nurse, visit	11.15
General practice assistant, visit	9.16
Outpatient clinic, visit	72.00
Complementary therapist	36.00
Occupational health specialist, visit	72.00
Abdominal ultrasound	86.20
Abdominal X-ray	52.20
Abdominal CT scan	235.90
Cystoscopy	395.50
Urodynamic test	395.50
Urinalysis	9.62
Urine culture	15.20
Blood test	12.90
Hospital admission, day	457.00
*Direct non-healthcare costs*	
Paid help, hour	As indicated by patient
Informal care, hour	12.50
Other patient costs	As indicated by patient
*Indirect costs*	
Absenteeism paid work, hour	Depending on age of patient

### Statistical analysis

The target number of 280 participants for this trial (140 in each arm) was based on an incorrect a priori sample size calculation and incorrect assumptions. A post hoc power calculation is presented here. When the sample size in each group is 150, a 2-group, 2-sided t test with an α of .05 will have 80% power to reject the null hypothesis that the mean number of annual symptomatic UTIs in the cranberry group is 1.3 greater than in the TMP-SMX group, assuming that the expected difference in means is 0 and the common SD is 4.0 (nQuery Advisor, version 7.0; Statistical Solutions Ltd, Cork, Ireland).

The statistical analyses were performed according to the intention-to-treat principle. Multiple Imputation (MI) as implemented in Stata 12 was used to impute missing cost and effect data for women who did not withdraw informed consent. An imputation model was created that contained variables that were related to missing data or the outcome measure, and variables that differed at baseline between the groups. Predictive mean matching was used to account for the skewed distribution of costs. By MI 20 imputed data sets were created, each of which was analyzed separately. The results of the 20 analyses were pooled using Rubin's rules [24].

Seemingly unrelated regression was used to estimate cost and effect differences between the groups while accounting for potential correlation between costs and outcomes [25]. Incremental cost-effectiveness ratios (ICERs) were calculated by dividing the difference in total costs between cranberries and TMP-SMX by the difference in clinical effects. Non-parametric bootstrapping with 5000 replications was used to estimate 95% confidence intervals around cost differences and the uncertainty surrounding the incremental cost-effectiveness and cost-utility ratios (5000 replications) [26].

The bootstrapped cost-effect pairs were plotted on a cost-effectiveness plane (CE plane) [27] and used to estimate cost-effectiveness acceptability curves (CEA curves). CE planes show the uncertainty around the ICER. On the x-axis the differences in effect are plotted and on the y-axis the differences in costs for all bootstrapped cost-effect pairs. The CE plane results in four quadrants. In the northeast (NE) quadrant, the intervention is more expensive and more effective than control. In the southeast (SE) quadrant, the intervention is less expensive and more effective than control and is said to dominate control. In the southwest (SW) quadrant, the intervention is less expensive and less effective than control. In the northwest (NW) quadrant, the intervention is more expensive and less effective than control and is said to be dominated by control. CEA curves show the probability that the intervention is cost-effective in comparison with the control treatment for a range of ceiling ratios. The ceiling ratio is defined as the amount of money society is willing to pay to gain one unit of effect [28]. For example, a ceiling ratio of 0 €/QALY indicates that society is not willing to pay anything to gain 1 QALY and a ceiling ratio of 20,000 €/QALY that society is willing to pay €20,000 to gain 1 QALY.

### Sensitivity analysis

Two sensitivity analyses were performed. In the first sensitivity analysis, costs of side effects of the treatments were also estimated (see appendix for included side effects and associated costs). In a second sensitivity analysis, costs of side effects and absenteeism were included. The number of hours of absenteeism from paid work due to UTI was multiplied with the number of UTIs in that month to estimate costs of absenteeism.

## Results

### Participant flow

From January 1, 2005, to August 31, 2007, 221 premenopausal women with recurrent UTIs were recruited: 111 were randomized to cranberries and 110 to TMP-SMX. Table 2 shows baseline characteristics of the participants. Although women in the TMP-SMX group were statistically significantly more likely to have ever used cranberries in the past than women in the cranberry group, there were no indications that there were problems with randomization. Therefore, we concluded that there were no relevant differences between the two intervention groups.

**Table 2:** Baseline characteristics.

Characteristic	Cranberry (n = 111)	TMP-SMX (n = 110)
Age, median (IQR), y	34.8 (22.8–44.4)	36.1 (26.9–46.3)
No. of UTIs in preceding year, median (IQR)	7 (4–11)	6 (4–8)
Anatomic/functional abnormalities of urinary tract	11 (9.9)	10 (9.1)
Medical history with urologic surgery	7 (6.3)	7 (6.4)
Catheter	5 (4.5)	3 (2.7)
Diabetes mellitus	3 (2.7)	1 (0.9)
Sexually active	99 (89.2)	104 (94.5)
Use of incontinence material	15 (13.5)	13 (11.8)
Use of antibiotic 3 mo before inclusion	85 (76.6)	86 (78.2)
Use of cranberries ever	84 (75.7)	96 (87.3)

Presented are N(%) unless indicated otherwise.

Abbreviations: IQR, interquartile range; TMP-SMX, trimethoprim-sulfamethoxazole; UTI, urinary tract infection.

**Figure 1:**
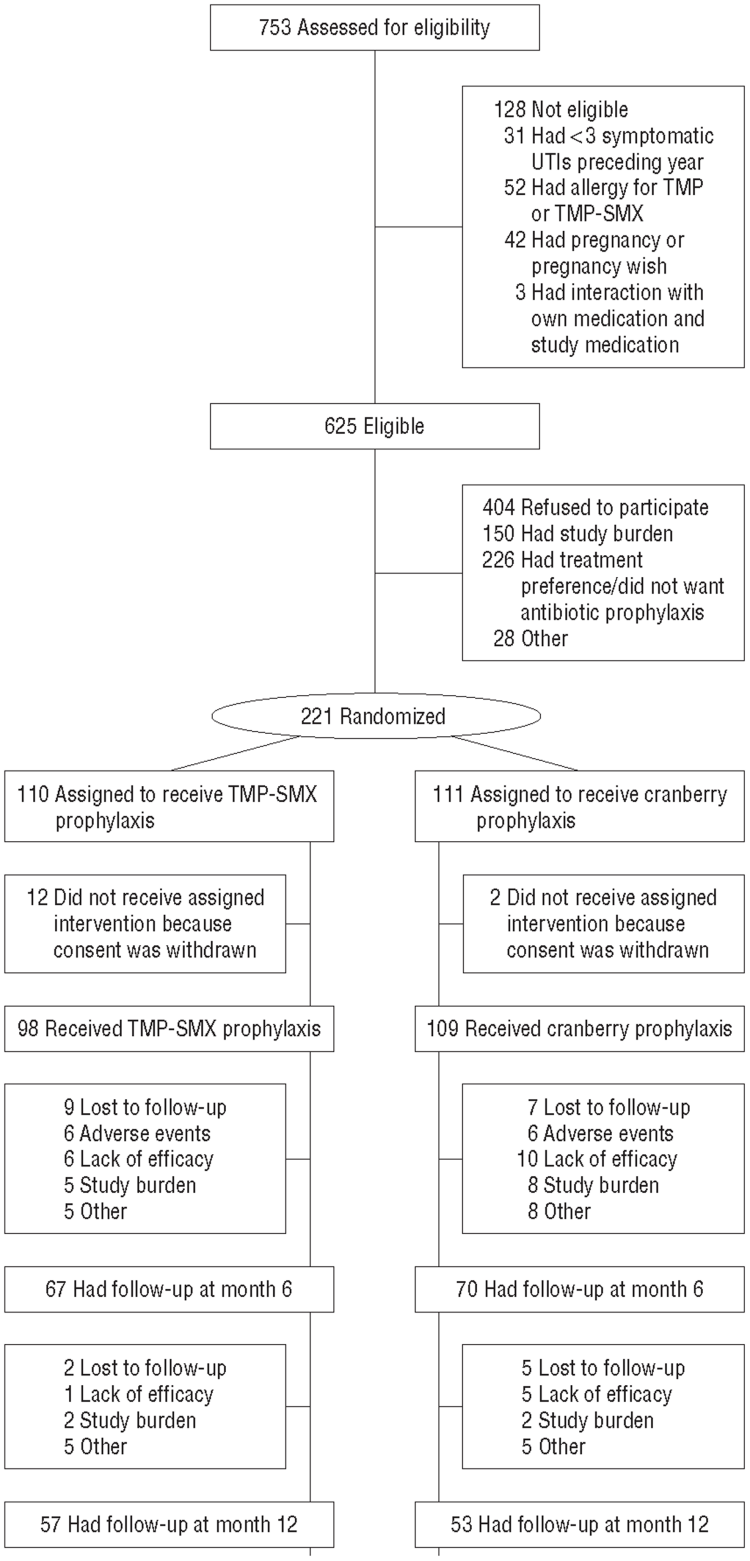
Flow of participants through the trial. The lack of efficacy was determined by the patients. Abbreviations: TMP-SMX, trimethoprim-sulfametoxazole.

The flow of participants is presented in Figure 1. After completing the baseline measurements, 2 women in the cranberry group and 12 women in the TMP-SMX group withdraw informed consent [15]. After 12 months, complete follow-up for costs and the primary outcome was available for 42 (38%) women in the cranberry group and 51 (46%) women in the TMP-SMX group. Complete follow-up on quality of life was available for 27 (24%) women in the cranberry group and 28 (25%) women in the TMP-SMX group. Women who did not complete all questionnaires were younger, had experienced more UTIs in the previous year, were more likely to have a catheter, were more likely to be sexually active, and were less likely to have used cranberries before.

There were no statistically significant differences between the cranberry and the TMP-SMX groups in the percentages of patients with any or a specific AE or a SAE. In the TMP-SMX group, 1 woman had a SAE (Stevens-Johnson syndrome), which led to her withdrawal. There were no SAEs in the cranberry group.

### Clinical outcomes

Table 3 presents the results of the analyses of clinical outcomes. After 12 months, the mean number of UTIs in the cranberry group was 4.3 and in the TMP-SMX group 2.7. Although there were considerably more UTIs in the cranberry group than in the TMP-SMX group, this difference was not statistically significant at the conventional threshold of 0.05 (mean difference 1.6, 95% CI −0.23 to 3.5). Outcomes for satisfaction with the prophylactic treatment and QALYs after 12 months were in line with the difference in UTIs. Women in the cranberry group were less satisfied than women in the TMP-SMX group and experienced less QALYs. However, 95% confidence intervals showed that these differences were not statistically significant.

**Table 3:** Multiply imputed pooled effects and costs (€, 2009) after 12 months.

Outcome	Cranberry (n = 109)	TMP-SMX (n = 98)	Difference (95% CI)
UTIs	4.3 (0.84)	2.7 (0.50)	1.6 (−0.23 ; 3.5)
Satisfaction	59 (4)	68 (3)	−9 (−19 ; 1)
QALYs	0.76 (0.03)	0.80 (0.02)	−0.04 (−0.10 ; 0.03)
Direct healthcare costs	571 (97)	348 (39)	223 (−3 ; 450)
Intervention costs	302 (12)	188 (7)	114 (87 ; 141)
Direct non-healthcare costs	56 (30)	30 (13)	26 (−49 ; 100)
Total costs	627 (96)	378 (42)	249 (70 ; 516)

Presented are mean (SEs) unless indicated otherwise.

Abbreviations: TMP-SMX, trimethoprim-sulfamethoxazole; UTI, urinary tract infection; QALY, Quality-Adjusted Life-Year.

### Costs

Costs (in Euros) in the cranberries and TMP-SMX group are also shown in Table 3. Direct healthcare costs were the greatest contributor to total costs. Within direct healthcare costs, intervention costs constituted approximately half of the total costs. Direct healthcare and non-healthcare costs in the cranberry group were higher than in the TMP-SMX group, but confidence limits were too wide to exclude no difference in costs. Total costs in the cranberry group were higher than in the TMP-SMX group (mean difference €249, 95% CI 70 to 516).

### Cost-effectiveness

**Figure 2:**
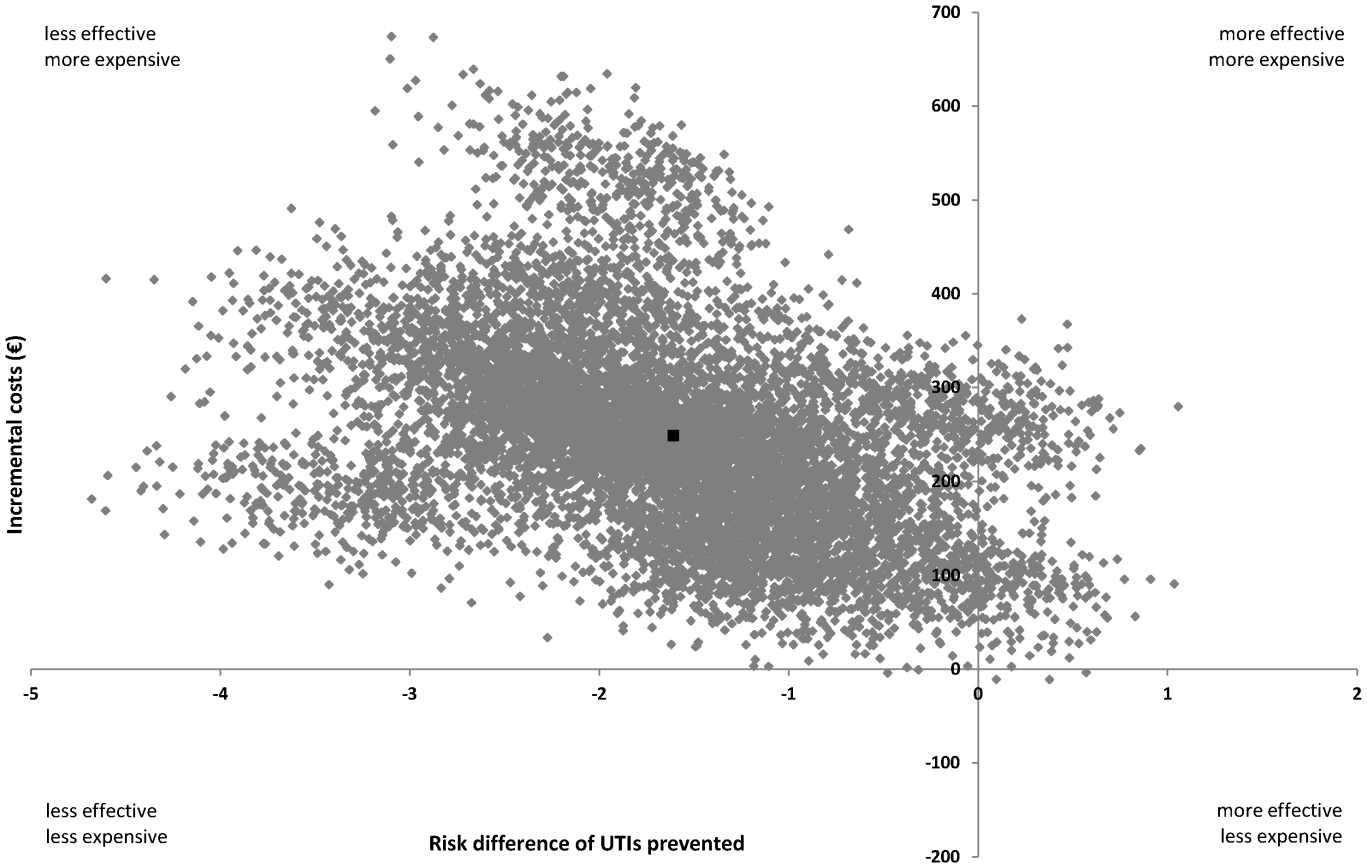
Cost-effectiveness plane for number of urinary tract infections prevented during 12 months (cranberry prophylaxis vs TMP-SMX prophylaxis). The black dot indicates the point estimate of the ICER (1.6 prevented UTIs less and €247 more costs in the cranberry group as compared to the TMP-SMX group) and the grey dots indicate the bootstrapped cost-effect pairs to reflect the uncertainty around the ICER. Abbreviations: ICER, Incremental Cost-Effectiveness Ratio; TMP-SMX, trimethoprim-sulfametoxazole; UTI, Urinary Tract Infection.

**Figure 3:**
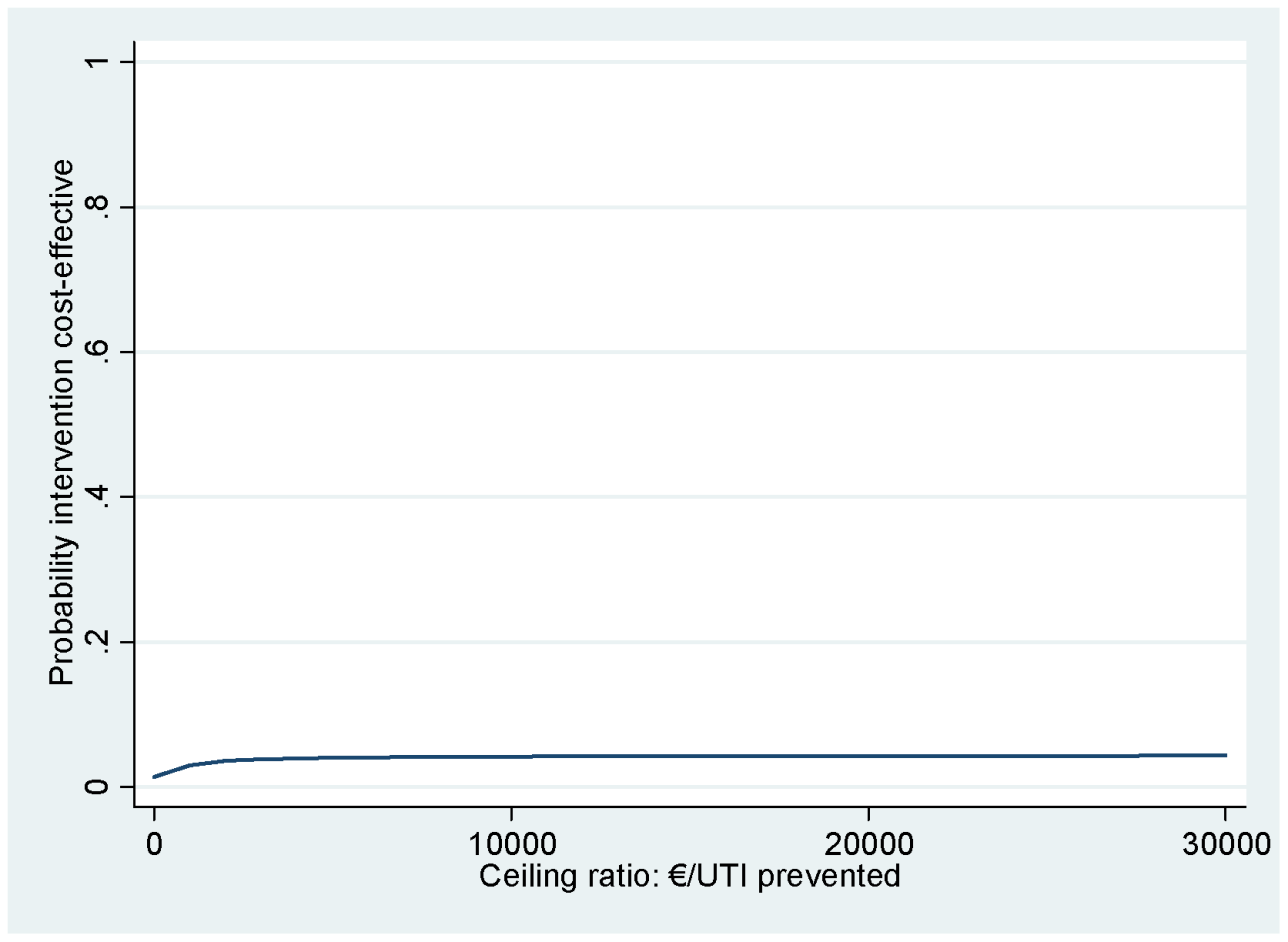
Cost-effectiveness acceptability curve for number of urinary tract infections prevented during 12 months (cranberry prophylaxis vs TMP-SMX prophylaxis). Abbreviations: TMP-SMX, trimethoprim-sulfametoxazole; UTI, Urinary Tract Infection.

The results of the cost-effectiveness and cost-utility analyses are presented in Table 4. The ICER for number of UTIs prevented was −156, meaning that 1 prevented UTI less in the cranberry group was associated with €156 higher costs as compared to the TMP-SMX group. The CE plane in Figure 2 shows that 96% of the cost-effect pairs are located in the NW quadrant (less effective and more expensive) indicating that prophylactic treatment with cranberries was dominated by prophylactic treatment with TMP-SMX. This was confirmed by the cost-effectiveness acceptability curve in Figure 3. For a ceiling ratio of 0 €/UTI prevented, the probability that prophylactic treatment with cranberries was cost-effective in comparison with prophylactic treatment with TMP-SMX was 0 (proportion of cost-effect pairs in the southern quadrants of the CE plane) and this slowly increased to 0.04 (proportion of cost-effect pairs in the eastern quadrants of the CE plane) as the ceiling ratio increases toward ∞ €/UTI prevented.

**Table 4:** Results of the cost-effectiveness and cost-utility analyses.

	Sample size					CE plane			
Analysis	Cranberry	TMP-SMX	Costs	Effects	ICER	NE	SE	SW	NW
*Main analysis*									
UTIs prevented	109	98	249 (70 ; 516)	−1.6 (−3.5 ; 0.23)	−155	4%	0%	0%	96%
Satisfaction	109	98	249 (70 ; 516)	−9 (−19 ; 1)	−27	3%	0%	0%	97%
QALYs	109	98	249 (70 ; 516)	−0.04 (−0.10 ; 0.03)	−8050	18%	0%	0%	82%
*With costs of side effects*									
UTIs	109	98	247 (56 ; 455)	−1.6 (−3.2 ; −0.04)	−154	4%	0%	0%	96%
Satisfaction	109	98	247 (56 ; 455)	−10 (−19 ; 0.27)	−26	3%	0%	1%	96%
QALYs	109	98	247 (56 ; 455)	−0.03 (−0.09 ; 0.02)	−7736	12%	0%	1%	87%
*With costs of side effects and absenteeism*									
UTIs	109	98	324 (113 ; 571)	−1.7 (−3.2 ; −0.16)	−194	1%	0%	0%	99%
Satisfaction	109	98	324 (113 ; 571)	−9 (−19 ; 1.8)	−37	5%	0%	0%	95%
QALYs	109	98	324 (113 ; 571)	−0.02 (−0.08 ; 0.03)	−15664	18%	0%	0%	82%
*Complete cases*									
UTIs	42	51	198 (109 ; 304	1.0 (0.07 ; 2.2)	−205	3%	0%	0%	97%
Satisfaction	42	51	198 (109 ; 304)	−6 (−18 ; 5)	−33	15%	0%	0%	85%
QALYs	27	28	86 (−59 ; 190)	0.001 (−0.06 ; 0.06)	82630	46%	6%	3%	45%

NE: treatment with cranberries is more effective and more expensive than treatment with TMP-SMX.

SE: treatment with cranberries is more effective and less expensive than treatment with TMP-SMX.

SW: treatment with cranberries is less effective and less expensive than treatment with TMP-SMX.

NW: treatment with cranberries is less effective and more expensive than treatment with TMP-SMX.

Abbreviations: TMP-SMX, trimethoprim-sulfamethoxazole; UTI, urinary tract infection; QALY, Quality-Adjusted Life-Year.

Results for satisfaction with the prophylactic treatment received were similar to the results for UTIs prevented. Cost-effectiveness acceptability curves show that for all ceiling ratios, the probability that prophylactic treatment with cranberries was cost-effective in comparison with prophylactic treatment with TMP-SMX was 0.03 at most. For QALYs, the cost-effectiveness acceptability curve showed that at a ceiling ratio of 0 €/QALY the probability that prophylactic treatment with cranberries was cost-effective in comparison with prophylactic treatment with TMP-SMX was 0 and that this probability slowly increased to 0.18 for ceiling ratios of ∞ €/QALY.

### Sensitivity analysis

The results of the sensitivity analysis including costs of side effects were similar to the main analysis. In this sensitivity analysis, the multiple imputation resulted in slightly higher mean estimates for direct healthcare costs in both groups as expected. Mean costs of side effects were €34 in the cranberry group and €39 in the TMP-SMX group. The difference in total costs was comparable to the main analysis (mean difference 247, 95% CI 56 to 455). Results of the cost-effectiveness analyses indicated that, despite these lower costs due to side effects in the cranberry group, prophylaxis with cranberries is not cost-effective compared to prophylaxis with TMP-SMX.

In the second sensitivity analysis, both costs of side effects and costs of absenteeism from paid work were included. Absenteeism costs were €61 in the cranberry group and €37 in the TMP-SMX group. The difference in total costs was somewhat larger than in the main analysis (mean difference €324, 95% CI 113 to 571). The cost-effectiveness analyses indicated that prophylaxis with cranberries is not cost-effective compared to prophylaxis with TMP-SMX.

## Discussion

### Main findings

In this study, the cost-effectiveness of prophylactic treatment with cranberries was evaluated in comparison with prophylactic treatment with TMP-SMX. The results showed that prophylactic treatment with cranberries was less effective than prophylactic treatment with TMP-SMX, although no effect could not be ruled out based on 95% confidence intervals. Costs in the cranberry group were higher than in the TMP-SMX group. Based on these results, prophylaxis with cranberries was not considered cost-effective compared to prophylaxis with TMP-SMX. In fact, the cost-effectiveness planes show that in most bootstrap samples, cranberry prophylaxis is dominated by TMP-SMX prophylaxis.

This economic evaluation was based on the same study as the previously published clinical paper by Beerepoot et al. [15] Due to the different imputation techniques, the number of symptomatic UTIs in the two treatment groups differs slightly between the two papers. However, in both papers there were considerably more UTIs in the cranberry group than in the TMP-SMX group.

Prophylactic treatment with cranberries was more expensive than prophylactic treatment with TMP-SMX. Also, the treatment costs of UTIs were higher in the cranberry group than in the TMP-SMX group, because there were more recurrent UTIs in the cranberry group. However, costs of the prophylactic treatment were the largest contributor to the difference in costs between the two groups. Antibiotics are relatively cheap, because patents have expired. Considering that the effects of cranberry prophylaxis are much smaller than those of TMP-SMX prophylaxis, prices of cranberry products must fall considerably before one may expect prophylactic treatment with cranberries to be cost-effective compared to TMP-SMX prophylaxis.

### Comparison with existing literature

A recent review by Vasileiou et al (2013) concluded that cranberry prophylaxis may be effective in comparison with placebo treatment [29]. However, this review included two studies evaluating the effectiveness of cranberry versus trimethoprim (TMP) [15], [30]. The study by McMurdo et al showed that more women in the cranberry group had a symptomatic UTI that was treated with antibiotics compared to the TMP group [30]. These findings are similar to the findings reported here and in the original clinical paper by Beerepoot et al. [15]

Stothers evaluated the cost-effectiveness of cranberry products in comparison with placebo. This study showed that cranberry products reduced the mean number of UTIs during a year in comparison with placebo, but at considerably increased costs [14].

### Strengths and limitations

A limitation of this study is that absenteeism from paid work due to UTI was not measured in a reliable manner. Women were asked how many hours they were absent from work in the past week due to UTI. Thus, we had to extrapolate this over the whole recall period of the questionnaire (1 month) by multiplying the number of UTIs with the number of hours that women were absent from work. Another limitation is the rate of missing data in this study. Only 38% of the women in the cranberry group and 46% of the women in the TMP-SMX group returned all 12 monthly questionnaires during the 12 months of follow-up. However, 61% of the women in the cranberry group and 71% of the women in the TMP-SMX group returned 10 or more questionnaires, indicating that the majority of the women returned 75% or more of the questionnaires. We tried to overcome this limitation by imputing missing cost and effect data with multiple imputation techniques. Multiple imputation is considered the most appropriate technique to deal with missing cost data, because it takes into account the uncertainty in the imputation [31], [32], [33]. The use of multiple imputation in this study can, therefore, be considered as an important strength. Another strength is that this is the first rigorously performed economic evaluation comparing cranberry prophylaxis with antibiotic treatment in women with recurrent UTIs. Stothers evaluated the cost-effectiveness of cranberry tablets and juice versus placebo, but that study did not include a formal statistical analysis of costs, effects and cost-effectiveness [14]. Other strengths include the double-blind design of this pragmatic RCT and the fact that women for this study were recruited from an outpatient population. This enhances the validity of our findings considerably.

### Implications for further research

This study did not take into account the incremental costs associated with antibiotic resistance. However, it is important to take this into account, because antibiotic resistance can lead to increased costs in two ways. First, expensive antibiotic treatments and even hospitalization may be needed to treat UTIs caused by resistant microorganisms. Second, resistant bacteria may spread to other individuals [34]. The clinical study accompanying this economic evaluation already showed high resistance rates after 1 month of prophylaxis with TMP-SMX [15]. A recent report estimated that infections due to multidrug-resistant bacteria in the EU result in extra healthcare and lost productivity costs of at least €1.5 billion each year [35]. Therefore, for future research, it is recommended to take the costs of antibiotic resistance into account. A decision-analytic model in which the probability of antibiotic resistance and the associated costs are mathematically modeled may be needed to evaluate the societal economic burden of antibiotic resistance in the prophylaxis of recurrent UTIs.

The optimum dosage of cranberry extract in vivo is not clear yet; a dose-finding study is under way (clinicaltrials.gov, Identifier: NCT00100061). It is possible that the dosage used in this study was too low and that effects of cranberry prophylaxis were underestimated. Therefore, future research should investigate the effects and cost-effectiveness of optimum dosages of cranberry prophylaxis. This is particularly important, because many women are afraid of contracting antibiotic resistant bacteria due to long-term prophylactic antibiotic treatment and, therefore, prefer no or non-antibiotic prophylaxis.

## Conclusions

In conclusion, this economic evaluation showed that UTI prophylaxis in women with recurrent UTIs with cranberries is not cost-effective in comparison with prophylaxis with TMP-SMX. Indeed, cranberry prophylaxis was less effective and more expensive than (dominated by) TMP-SMX. However, some caution should be used when interpreting this result considering the large rate of missing data.

Within the framework of this randomized trial, it was not possible to take into account costs attributed to increased antibiotic resistance although this may have an important effect on the cost-effectiveness estimates. Future modeling studies are recommended to investigate the impact of costs of increased antibiotic resistance. Moreover, although we based the dosage of cranberry extract on available evidence, this may not be the optimal dosage. Results may change when this optimal dosage is identified.

## Supporting Information

Checklist S1 **CONSORT checklist.**
(DOC)

Protocol S1 **Trial protocol.**
(DOC)

Table S1 **Included side effects and associated costs.**
(DOCX)
