# Supplementary material for: Cost-Effectiveness of Cranberries vs Antibiotics to Prevent Urinary Tract Infections in Premenopausal Women: A Randomized Clinical Trial
Source: PLoS One. 2014 Apr 4;9(4):e91939. doi: 10.1371/journal.pone.0091939 (PMC3976255; doi:10.1371/journal.pone.0091939)
Supplement: Protocol S1 — Trial protocol. (DOC) [file pone.0091939.s002.doc]

**Non-antibiotic versus Antibiotic Prophylaxis for Recurrent Urinary Tract Infections (NAPRUTI)**

**Protocol**

**Final Version (03/05/2005)**

**Principal Investigators**

Dr. S.E. Geerlings (woman)

Function: internist-infectiologist

Academic Medical Center

Infectious Diseases, Tropical Medicine & AIDS

Meibergdreef 9

1105 AZ Amsterdam

The Netherlands

Phone: +31-20-5664380

Fax: +31-20-6972286

E-mail: [S.E.Geerlings@amc.uva.nl](mailto:S.E.Geerlings@amc.uva.nl)

Dr. E.E. Stobberingh (woman)

Function: medical microbiologist

Academic Hospital Maastricht

Medical Microbiology

P. Debyelaan 25

6202 AZ Maastricht

The Netherlands

Phone: +31-43-3874644

Fax: +3143-3876643

E-mail: [est@lmib.azm.nl](mailto:est@lmib.azm.nl)

**Study coordinator**

Drs. M.A.J. Beerepoot (woman)

Function: PhD-student, MD

Academic Medical Center, Amsterdam

Infectious Diseases, Tropical Medicine & AIDS

Phone: +31-20-5667921

Fax: +31-20-6972286

E-mail: [M.A.Beerepoot@amc.uva.nl](mailto:S.E.Geerlings@amc.uva.nl)

**Other members study group**

Dr. C. de Borgie (woman)

Function: epidemiologist

Academic Medical Center, Amsterdam

[c.deborgie@amc.uva.nl](mailto:c.deborgie@amc.uva.nl)

Dr. G. ter Riet (man)

Function: epidemiologist

Academic Medical Center, Amsterdam

[G.TerRiet@amc.uva.nl](mailto:G.TerRiet@amc.uva.nl)

Dr. T.M. de Reijke (man)

Function: urologist

Academic Medical Center, Amsterdam

[t.m.dereyke@amc.uva.nl](mailto:t.m.dereyke@amc.uva.nl)

Drs. S. Nys (woman)

Function: PhD-student

Academic Hospital Maastricht, Maastricht

snij@lmib.azm.nl

Dr. A. Verbon (woman)

Function: internist-infectiologist

Academic Hospital Maastricht, Maastricht

averb@lmib.azm.nl

Drs. A.Vyth (man)

Function: trial-pharmacist

Academic Medical Center, Amsterdam

a.vyth@amc.uva.nl

Dr. C. van de Beek (man)

Function: urologist

Academic Hospital Maastricht, Maastricht

Cvb@groupwise.azm.nl

Prof. Dr. P. Bindels (man)

Function: general practitioner and head of Research Department of General Practice

Academic Medical Center, Amsterdam

P.J.Bindels@amc.uva.nl

Dr. T. van Merode (woman)

Function: general practitioner

Academic Hospital Maastricht, Maastricht

[Tiny.vanMerode@hag.unimaas.nl](mailto:Tiny.vanMerode@hag.unimaas.nl)

Dr. G. Reid (man)

Function: urologist and director of the Canadian Research and Development Center for Probiotics

University of Western Ontario, London, Canada

gregor@uwo.ca

Dr. W. Slits (man)

Function: nursing home practitioner

Nursing Home Klevarie, Maastricht

w.slits@stichtingba.nl

Prof. dr. C.M.J.E. Vandenbroucke-Grauls (woman)

Function: medical microbiologist and head of the Laboratory of Medical Microbiology

Academic Medical Center, Amsterdam

[C.M.Vandenbroucke@amc.uva.nl](mailto:C.M.Vandenbroucke@amc.uva.nl)

**Study coordinators in participating centers**

Dr. G. van Andel (man)

Function: urologist

Onze Lieve Vrouwe Gasthuis, Amsterdam

g.vanandel@olvg.nl

Drs. E. van Haarst (man)

Function: urologist

Sint Lucas Andreas Hospital, Amsterdam

[e.vanhaarst@slaz.nl](mailto:e.vanhaarst@slaz.nl)

Drs. G.J. Hemmes (woman)

Function: geriatrician

Westfries Gasthuis, Hoorn

g.j.hemmes@westfriesgasthuis.nl

Prof. Dr. I.M. Hoepelman (man)

Function: internist-infectiologist

University Medical Center, Utrecht

[i.m.hoepelman@azu.nl](mailto:i.m.hoepelman@azu.nl)

Drs. J. Koeijers (woman)

Function: MD

Academic Hospital Maastricht, Maastricht

[J.koeijers@planet.nl](mailto:J.koeijers@planet.nl)

Drs. S.E. de Rooij (woman)

Function: geriatrician

Academic Medical Center, Amsterdam

s.e.derooij@amc.uva.nl

Dr. J.V. Zambon (man)

Function: urologist

Hospital Maasland, Sittard

[V.Zambon@orbisconcern.nl](mailto:V.Zambon@orbisconcern.nl)

Drs. J.P.C.M. van Campen (man)

Function: geriatrician

Slotervaart Ziekenhuis, Amsterdam

Gejvc@slz.nl

**Cooperation**

Springfield Nutraceuticals

Function: delivery cranberry capsules (Cran-Max®) and placebos

Simon Stevinstraat 15

3261 MG Oud-Beijerland

The Netherlands

Phone: +31-(0)186-626173

Contact persons: S. Bleijswijk (woman, scientific nutritionist) [saskia@springfieldnutra.com](mailto:saskia@springfieldnutra.com)

P. F. Hogerhuis (man, managing director)

[sf-peter@springfield.nl](mailto:sf-peter@springfield.nl)

Chr. Hansen Function: delivery of *Lactobacillus* capsules and matching placebos Bøge Allé 10-12
DK-2970 Hørsholm Denmark
Phone: +45 45747474
Contact person: N. P. Bak (man, marketing manager, human health & nutrition) [NielsP.Bak@dk.chr-hansen.com](mailto:NielsP.Bak@dk.chr-hansen.com)

Tiofarma B.V.

Function: delivery of co-trimoxazolee tablets and matching placebos

Benjamin Franklinstraat 9

3261 LW Oud-beijerland

The Netherlands

Phone:0186-614778

Contactpersoon: J. Tio (man, pharmacist)

[jtio@tiofarma.nl](mailto:jtio@tiofarma.nl)

**Table of contents**

**1 Introduction 9**

1.1 Background 9

1.2 Rationale 9

1.3 Purpose 10

**2 Study objectives and endpoints 11**

2.1 Study objectives 11

2.1.1 Primary objectives 11

2.1.2 Secondary endpoints 11

2.2 Study endpoints 11

2.2.1 Primary endpoints 11

2.2.2 Secondary endpoints 11

[**3** **Methodology**](#__RefHeading___Toc70136200)  **12**

[3.1 Study design](#__RefHeading___Toc70136201)  12

[3.2 Study duration](#__RefHeading___Toc70136202)  12

3.3 [Randomization and blinding 12](#__RefHeading___Toc70136205)

3.3.1 Method of randomization 12

3.3.2 Method of blinding 12

3.3.3 Checking the success of double-blind conditions 13

[**4**](#__RefHeading___Toc70136217) **Study population 14**

4.1 Recruitment and screening method 14

4.2 Inclusion and exclusion criteria 14

4.2.1 Inclusion criteria 14

4.2.2 Exclusion criteria 14

4.3 Withdrawal from study 15

[**5**](#__RefHeading___Toc70136249) **Study Medication 16**

5.1 Choice of drug 16

5.1.1 Antibiotic prophylaxis 16

5.1.2 Non-antibiotic prophylaxis 16

5.2 Drug regimen, administration and duration 15

5.3 Drug formulation, supply and distribution 17

5.4 Concomitant medication 17

5.5 Compliance 17

**6 Clinical and laboratory evaluations 18**

6.1 Clinical evaluations 18

6.2 Laboratory evaluations 18

**7 Safety 20**

7.1 Adverse events 20

7.2 Stopping rules 20

7.3 Annual safety rapport 20

**8 Statistical analysis 21**

8.1 Power ans sample size calculations 21

8.2 Data analysis 21

8.2.1 General approach 21

8.2.2 Primary outcome 21

8.2.3 Secondary outcome 21

**9 Economic evaluation 23**

**10 Ethical considerations 25**

10.1 Medical Ethics Commitee 25

10.2 Informed consent 25

10.3 Subject confidentiality 25

10.4 Study modification/discontinuation 25

10.5 Benefits and risks 25

[**11 Publication policy**](#__RefHeading___Toc70136254)  **26**

[**12** **References**](#__RefHeading___Toc70136255)  **27**

**Appendix I : List of definitions 29**

**1 Introduction**

- 1. **Background**

Urinary tract infections (UTIs) occur frequently. The incidence of UTIs in a Dutch family doctor’s practice is about 30 to 40 per 1000 patients per year. Eighty to ninety percent of UTIs affect women (1). One in three women will have at least one UTI by the age of 24 years, and 40-50% of women will experience at least one UTI during their lifetime. The frequency of UTIs increases with age while, for example, patients with a urinary catheter, diabetes mellitus, spinal cord lesions, or a neurogenic bladder are particularly susceptible (2). Due to the increasing life expectancy and the increasing number of patients suffering from diabetes, it is to be expected that the prevalence of UTIs will rise (3).

In addition to the impact on quality of life, UTIs have financial implications (3;4). Although the costs associated with a single episode of UTI are relatively low, due to the high incidence total costs are estimated to be around 100 to 300 million Euro per year in the Netherlands (5).

It is of note that 20 to 30 percent of the women experiencing an episode of UTI will experience recurrent UTIs. Recurrent UTIs (RUTIs) are usually defined as at least three episodes of UTI in the previous twelve months or two episodes in the previous six months(6). RUTIs can be based on a “relapse” after treatment cessation with the pre-therapy isolate or as a “reinfection” with a different organism after initial treatment cessation (2). Long-term low-dose antibiotic prophylaxis can be recommended for prevention of reinfections in women with UTIs (7).

- 1. **Rationale**

Randomized trials (RCTs) have documented that continuous prophylaxis with trimethoprim-sulfamethoxazole (TMP-SMX) diminishes recurrences by 95 percent (from 2 to 3 episodes per patient-year to 1 or 2 episodes per 10 patient-years) (8). However, these successes may not last another decade now that antibiotic resistance is increasingly becoming a problem. The most important risk factor for having a UTI resistant to TMP-SMX is its recent or present use (9). During a 2-week follow-up study in healthy students, who took TMP or TMP-SMX prophylaxis, a total number of 82 out of 86 students developed resistant micro-organisms in their faeces (10).

It is known from clinical practice that many women are afraid of getting resistant bacteria. For that reason they do not want to take antibiotic prophylaxis over an extended period of time. Furthermore, antibiotics can cause adverse side effects. Therefore, the need for alternatives to antibiotic UTI-prophylaxis is evident. Knowledge of the pathogenesis of UTIs is essential to prevent infection from a small number of microbes which originate from outside the urinary tract (primary prophylaxis). Two essential steps in the pathogenesis of UTI are: colonization and adherence of uropathogens.

### UTIs result almost exclusively from the ascending route. Bacteria colonizing the perineum and vagina can enter the urethra and further ascend to the bladder or even the kidneys. The colonization can be inhibited by the normal vaginal flora: lactobacilli*,* which produce lactic acid, and maintain a low vaginal pH that inhibits the growth of many uropathogens. In postmenopausal women lactobacilli disappear. This results in a higher chance of colonization of the vagina with *Enterobacteriaceae*. It has been demonstrated that vaginal colonization with *Escherichia coli* and the absence or low levels of vaginal lactobacilli were independently associated with a history of a recent UTI (11). It has also been shown that oral administration of *Lactobacillus rhamnosus GR-1* and *L. reuteri RC-14* restores normal vaginal lactobacilli flora and reduces colonization by potential pathogenic bacteria (12). In a RCT with postmenopausal women with a history of recurrent UTIs, the incidence of UTIs was reduced in the estriol treated group compared to the placebo group (0.5 *versus* 5.9 episodes per patient year; p<0.001). More specifically, the topical application of vaginal estriol cream in the treatment group led to re-colonization of the vagina by lactobacilli in 23 of the 36 women. There was a strong positive relation between vaginal colonization status and the risk of infection: No UTIs developed in 20 of the 23 women who were colonized with lactobacilli (success rate of 87 percent) compared with 6 out of the 13 (success rate 46%; difference 41 (95%CI from 10 to 71) who were not colonized with lactobacilli (13).

### The adherence of uropathogens to the bladder mucosa is another essential step in the pathogenesis of UTIs. In that process, adhesins (fimbriae) are important virulence factors. Although virulence factors have been characterized best in *Escherichia coli* (the most prevalent uropathogen) many of the same principles are applicable to other Gram-negative uropathogens. Cranberries contain fructose and proanthocyanidine, which inhibit the adherence of type 1 fimbriae of *E. coli* (the most important virulence factor in cystitis) and the expression of P fimbriae (the most important virulence factor in pyelonephritis), respectively (14;15). In a RCT with pre-menopausal women, the incidence of UTIs in the cranberry-treated women was only 16% (versus 36% in the control group; difference 20%; 95%CI from 3% to 36%; p=0.023), during a 6 months follow-up (16). Furthermore, it has been demonstrated that cranberry juice may also offer protection against resistant strains of *E. coli* by a mechanism that is not likely to increase selective pressure associated with antibiotic resistance (17).

- 1. **Purpose**

To quantify the effect of antibiotic prophylaxis compared to two non-antibiotic prophylaxis modalities on the incidence of symptomatic UTIs in pre- and post-menopausal women with recurrent UTIs. In addition, we assess differences in the development of bacterial drug resistance.

**2 Study objectives and endpoints**

**2.1 Study objectives**

**2.1.1 Primary objectives**

- To determine if non-antibiotic prophylaxis is a non-inferior treatment in the prevention of recurrent symptomatic UTIs compared to the currently standard antibiotic prophylaxis in pre- and post-menopausal women (non-inferiority trial)
- To assess if non-antibiotic prophylaxis reduces the selection of antibiotic resistant micro-organisms in urine and faeces compared to currently standard antibiotic prophylaxis in pre- and postmenopausal women
  - 1. **Secondary objectives**
- To evaluate if non-antibiotic prophylaxis compared to antibiotic prophylaxis in pre- and post-menopausal women lead to differences in the:

- incidence of asymptomatic bacteriuria

- incidence of other (non-urinary tract) infections

- incidence of adverse events

- quality of life

- To evaluate the costs and cost-effectiveness of non-antibiotic prophylaxis compared to antibiotic prophylaxis in the prevention of recurrent UTIs in pre- and post-menopausal women.

**2.2 Study endpoints**

**2.2.1 Primary endpoints**

- the numbers of recurrences of symptomatic UTI

- time to first occurrence of antibiotic resistance in urine or

faeces

**2.2.2 Secondary endpoints**

- incidence of asymptomatic bacteriuria

- incidence of other infections

- adverse events

- quality of life

- costs per prevented UTI

**3 Methodology**

- 1. **Study design**

The NAPRUTI study consists of two trials: RCT-A and RCT-B. Premenopausal women will be included in RCT-A, postmenopausal women in RCT-B. Both trials are multi-center, randomized double blind clinical trials with a follow-up time of 15 months.

Both trials consist of two parts: part one deals with UTI recurrence (non-inferiority part); part two aims to show a difference with respect to the development of bacterial drug resistance.

- 1. **Study duration**

Both trials will recruit 280 women. Patient enrolment is anticipated to last 2 years. After randomization patients will be followed for 12 months during which they will receive study medication and 3 months afterwards (“wash-out”-period). Total study duration will be 48 months.

**3.3 Randomization and blinding**

**3.3.1 Method of randomization**

In both trials, computer-aided and concealed block randomization with pre-stratification by center and presence of a uncomplicated or complicated UTI will be performed. The Coordinating Center (AMC) prepares drug randomization lists for each study site in advance. All eligible and consenting patients will be randomly allocated to either:

- antibiotic prophylaxis (RCT A and B)

- non-antibiotic prophylaxis: cranberries (RCT-A),

lactobacilli (RCT-B)

- - 1. **Method of blinding**

In each trial two active compounds will be compared. Antibiotics are produced as tablets and non-antibiotics as capsules. Therefore, the “double-dummy”-method is used. Each patient receives 1 tablet and 2 capsules daily, but only one of them (the tablet or the capsules) contains the active substance. Drug packages are labelled with the Study Identification code (SID) by a central pharmacy. Afterwards packages are distributed to the hospital pharmacies of the participating centers. Neither the blinded study team nor the patient will be informed of the treatment until the data collection phase has been completed.

**Checking the success of double-blind conditions**

It is well known that double blinding is easier to strive for than to achieve. Therefore, at 12 months we ask each patient and the study coordinator which drug they think was administered.

**4 Study population**

- 1. **Recruitment and screening method**

Seven Dutch hospitals participate; three university hospitals (Academic Medical Center/Amsterdam, Academic Hospital Maastricht/Maastricht, University Medical Center/Utrecht) and four non-university hospitals (Onze Lieve Vrouwe Gasthuis/Amsterdam, Sint Lucas Andreas Hospital/Amsterdam, Maasland Hospital/Sittard, and Westfries Gasthuis/Hoorn). Urologist, infectious disease specialists or geriatricians from the above-mentioned centers determine patient eligibility and seek informed consent. The specialist will pass contact information from all eligible patients to the study coordinator. The specialist also gives an information letter and two informed consent forms (with one self-addressed envelope) to the patient. Afterwards, the study coordinator contacts the patient and checks if the patient really fulfils the entry criteria. Then the patient will be asked for informed consent. After informed consent the patient has to give or send one signed informed consent form back to the study coordinator, the other stays with the patient.

**4.2 Inclusion criteria and exclusion criteria**

**4.2.1 Inclusion criteria:**

- Women aged 18 years or older
- At least 3 symptomatic urinary tract infections, uncomplicated or complicated (for definitions see Appendix), in the year preceding study inclusion
  - 1. **Exclusion criteria:**

- Life expectancy </= 1 year

- Legally incapable

- A renal transplant in the medical history

- Contraindications for or

relevant interactions with co-trimoxazole (see Table 1)

RCT-A;

- Breastfeeding, pregnancy, or pregnancy wish for the next year

- Contraindications for or relevant interactions with cranberries (see Table 1)

Interactions of patient’s medication with study medication will be evaluated per patient. From each interaction the clinical relevance (risk for a particular patient) will be determined and the study coordinator will deliberate, along with the treating physician, in which way the interaction can be handled best.

Table 1, contra-indications and interactions with co-trimoxazole and cranberries.

| Study medication | Contra-indication | Interaction |
| --- | --- | --- |
| co-trimoxazole | allergy for sulfonamides and/or trimethoprim, severe renal or liver failure ( creatinin *or* ASAT and/or ALAT 5 times the upper limit) serious blood disorders, G6PD-deficiency, acute porphyria | oral anticoagulants, diuretics, sulfonylureumderi-vates, digoxin, methotrexate, fenytoin, procainamid, cyclosporine, folic acid antagonists, para-aminobenzoic acid and derivates |
| cranberries | known renal stones | oral anticoagulants |

If an eligible patient is using or has used cranberries, antibiotics, or estrogens (except oral contraceptives) in the 2 weeks in advance of screening, the patient is offered a “wash-out”-period before randomization.

**4.3 Withdrawal from study**

Following enrolment, participants may discontinue study participation for the

following reasons:

- Voluntary withdrawal.

- Withdrawal requested by one of the investigators or the treating physician.

Strict distinction is made between withdrawal of study medication (non-adherence) and willingness to participate in data collection efforts. This approach will likely reduce the number of missing data values and facilitate a proper intention-to-treat analysis.

**5 Study Medication**

**5.1 Choice of drugs**

**5.1.1 Antibiotic prophylaxis**

It has been recommended to treat complicated UTIs with antimicrobials which achieve high tissue concentrations, such as trimethoprim-sulfamethoxazole (TMP-SMX or co-trimoxazole). Furthermore, TMP-SMX is also a good prophylactic for patients with a history of uncomplicated recurrent UTIs (8). Therefore, we want to administer TMP-SMX to the study participants.

- - 1. **Non-antibiotic prophylaxis**

In postmenopausal women lactobacilli are often absent. There is a strong positive relation between vaginal-colonization status and the risk of UTI. Therefore, we will use lactobacilli to prevent UTIs in postmenopausal women. We will use *Lactobacillus rhamnosus GR-1* and *L. reuteri RC-14.* Both possess properties that are required to colonize the vagina and inhibit uropathogens.

Premenopausal women will receive cranberry capsules as non-antibiotic prophylaxis. Because of problems with compliance and adherence to therapy with cranberry juice (18), we will use cranberry capsules for this study. Since premenopausal women have normal concentrations of vaginal lactobacilli, it makes no sense to give them additional lactobacilli capsules.

Detailed information about product characteristics and evidence of safety will be described in a separate supplement (Investigational Medicinal Product Documentation).

- 1. **Drug regimen, administration and duration**

RCT-A (premenopausal women). Women in the index group receive one cranberry capsules of 500 mg (in the morning and at bedtime) and one placebo tablet co-trimoxazole of 480 mg (ante noctum). Women in the reference group receive one tablet co-trimoxazole of 480 mg (ante noctum) and one placebo cranberry capsule (in the morning and ante noctum).

RCT-B (postmenopausal women): Women in the index group receive one capsule containing *Lactobacillus rhamnosus GR-1* and *L. reuteri RC-14 (>1*0e9 CFU) (in the morning and ante noctum) and one placebo tablet co-trimoxazole of 480 mg (ante noctum). Women in the reference group receive one tablet co-trimoxazole of 480 mg (ante noctum) and a placebo lactobacillus capsule (in the morning and ante noctum). Thus, in both trials each woman receives once a day one tablet and twice daily one capsule. All capsules and tablets must be taken by mouth for the duration of one year.

- 1. **Drug formulation, supply and distribution**

Cranberry capsules (Cran-Max®) and matching placebos identical in appearance to the cranberry capsules will be obtained from Springfield Nutraceuticals, Oud-Beijerland, the Netherlands. Each active capsule contains 500 milligram of cranberry extract.

The lactobacilli for the lactobacillus capsules (*Lactobacillus rhamnosus GR-1* and *L. reuteri RC-1),* were grown in bulk fermentation vessels under European Approved Good Manufacturing Practice by Chr. Hansen, Denmark. The organisms are freeze- dried and placed in gelatine capsules, with dosage at 10e9 per capsule. Chr. Hansen will also deliver lactobacillus placebos identical in appearance to the capsules with two strains of lactobacilli.

Tablets of co-trimoxazole 480 mg and placebos identical in appearance to the co-trimoxazole tablets are delivered by Tiofarma B.V., Oud-Beijerland, the Netherlands.

Three-monthly, patients receive a package with study medication from the pharmacy in the nearest participating center. Each package is labelled with a SID and contains both the active compound as well as the placebo.

- 1. **Concomitant medications**

Concomitant medication, as listed in table 1, may cause interactions while on study. The general practitioner (GP) will be informed about the participation in the NAPRUTI study. Hence, the GP can take this into account when prescribing drugs. Also patients will receive a card, with relevant study information. They can show it to other healthcare personnel as required.

**5.5 Compliance checking method**

The compliance will be measured monthly (together with the collection of the vaginal swabs and urine and faeces samples) by pill counts. In addition, vaginal and faecal cultures will be examined on the presence of lactobacilli, and urine concentrations of TMP-SMX and proanthocyanidins will be determined.

1. **Clinical and laboratory evaluations**
   1. **Clinical evaluations**

During the course of the study, a set of Case Report Forms (CRFs) is completed. At t= 0 (at inclusion) demographic data (age, place of residence [for example nursing home], sexual activity, relevant medical history and concomitant diseases, number of UTIs in the previous year, medication, known urinary tract abnormalities, urinary catheter use) will be collected by the study coordinator.

Each month thereafter, during the 15 months of follow up, a questionnaire is sent to the patient. On those questionnaires, other relevant parameters concerning possible adverse events (e.g., allergic reactions, skin rash, gastrointestinal disturbances), possible interactions with other medication, and the medical history (change of place of residence, new concomitant diseases, medication change, pregnancy, hospital admissions, invasive procedures, operations, urinary catheter placement or removal) during the follow-up will be noted.

Quality of life questionnaires (concerning the general well-being and related to the disease) will be administered at baseline and 6, 12 and 15 months post-randomization.

- 1. **Laboratory evaluations**

At t= 0 and monthly thereafter, during the one year intervention period and the three months follow-up after stopping the intervention, material for culture will be sent to the patients. Vaginal swabs as well as samples from urine and faeces have to taken each month. Urine samples and faecal samples will be quantitatively analysed for the presence and degree of antibiotic (TMP-SMX) resistant micro-organisms. In addition, the vaginal swabs (2x) will be examined on the presence of lactobacilli and to one of the Gram stained swabs a Nugent score will be given. The scoring system examines vaginal cells at random and applies a 0-3 score for normal (dominated by Gram-positive bacilli resembling lactobacilli), 4-6 for intermediate (lactobacilli present along with Gram-variable rods), and 7-10 for bacterial vaginosis (no lactobacilli seen and cells colonized by Gram-negative rods). Urine samples will be examined for the presence of proanthocyanidins.

All samples (urine, faeces and vaginal swabs) will be analysed in the microbiology laboratory of the University Hospital Maastricht. To prevent growth of uropathogens in urine samples during transportation, dipslides will be used: a dipslide will be dipped in a fresh voided urine sample according to the manufacturer’s instructions. Dipslide, vaginal swabs (in transport medium) and faecal samples (1-2 gram) will be sent to the microbiological laboratory in Maastricht. The antibiotic susceptibility of the isolated resistant micro-organisms in the urine and faecal samples will be determined as a microbroth or Agar dilution method according to the SWAB standard [see Resistentie surveillance standaard SWAB 1999]. All other infections during the intervention and the follow-up period will be registered. Only in case of a symptomatic UTI, the patient will be asked to send a urine-dipslide to the microbiological laboratory in Maastricht.

For details about specimen collection requirements and laboratory tests: see table 2.

| **Specimen Type** | **When** | **Determination** | **Test** | **(Interpretation of)**  **results** |
| --- | --- | --- | --- | --- |
| Urine | Baseline, each month during follow-up + in case of symptomatic UTI | - Culture (max. 2 m.o.)  - Concentration TMP-SMX  - Analysis TMP-SMX -resistant m.o.  - Concentration proanthocyanidins | Dipslide  Microbroth dilution | Positive: >/=10³ CFU/ml urine  R, S, MIC (criterions NCCLS) |
| Faecal sample | Baseline, each month during follow-up | - Concentration *E.coli*  - Analysis TMP-SMX -resistant m.o.  - Concentration total lactobacilli, *L.GR-1, L. RC-14* | Agar dilution  Quantative agar culture | CFU/gram  R, S, MIC (criterions NCCLS)  CFU/gram |
| Vaginal swab | Baseline, each month during follow-up | - Concentration  total lactobacilli, *L.GR-1, L. RC-14* | Quantative agar culture | CFU/ml |

Table 2, specimen collection requirement and laboratory tests. M.o = micro-organisms, TMP-SMX = trimethoprim-sulfamethoxazole, CFU = colony forming units, R = resistant, S = sensitive, MIC = minimum inhibitory concentration, *L.GR-1* = *Lactobacillus rhamnosus Gr-1, L. RC-14* = *Lactobacillus reutri RC-14.*

**7 Safety**

**7.1 Adverse event**

As described above, all patients will be checked for contra-indications (including renal and liver failure) and relevant interactions for or with the drugs used in this study. Patients with contra-indications for or relevant interactions with study medication will be excluded from the study.

Most reported adverse events from the used antibiotics are: gastro-intestinal disturbances, vaginal symptoms (rash or itching) and allergic skin reactions. Because of the low prescribed dosage of antibiotics, and frequent evaluations, major adverse events are expected to occur very rarely and will be detected early.

The safety of cranberries is considered to be excellent. Some patients may experience a slight laxative effect, depending on the amount ingested (19). Nevertheless, at least one author warned that ingesting a large amount of cranberries over a longer period of time may increase risk of some types of urinary stones in high-risk patients because of the increased urinary excretion of oxalate and slight urinary acidification (20). There are also reported several cases about the possible interaction between cranberries and warfarin (21). For those reasons patients with a history of nephrolithiasis or those who use oral anticoagulants are excluded from RCT-A.

In the few studies using *Lactobacillus rhamnosus GR-1* and *L. reuteri RC-14* no adverse events were reported after oral intake (12).

An adverse event (AE) is defined as any health-related reaction, effect, toxicity or abnormal laboratory result that a participant experiences during the course of a study irrespective of relationship to study treatment. This includes changes in a participant's condition or laboratory results which have or could have a deleterious effect on a participant's health or well-being.

A serious adverse event is defined as any experience that is fatal or life-threatening, permanently disabling, requires in-patient hospitalization, or is otherwise judged to be serious by the study coordinator.

**7.2 Stopping rules**

The study drug may be withheld or discontinued permanently if the AE is thought to be possibly related to the study drug, as judged by the patient’s general practitioner or the study coordinator.

- 1. **Annual safety rapport**

Each year an annual safety rapport will be written. This is a safety report taking into account all new safety information received during the reporting time. This report will be examined by a group of independent medical specialists.

**8 Statistical analysis**

**8.1 Power and sample size calculations**

### Non-inferiority part: It is known from clinical practice that many women are afraid of contracting resistant bacteria. For that reason they do not want to take antibiotic prophylaxis over an extended period of time. These women are even willing to take non-antimicrobial prophylaxis with a lower success rate in preventing UTIs compared to antimicrobial prophylaxis. Therefore, we accept a 10 percent lower success rate of the non-antibiotic compared to the antibiotic prophylaxis.

### Based on an expected success rate in prevention of recurrent UTIs with TMP-SMX of 95% [2], measured success rates of 85% and 87% in the premenopausal women treated with cranberries [8], and postmenopausal women treated with lactobacilli [5], respectively, a type I error rate of 0.05 (alpha, one-sided) and a type II error rate of 0.20 (beta), 130 analysable women are needed for each of the four treatment arms.

### Resistance part: For both trials, the power to detect a 15% difference in prevalence of TMP-SMX resistance (30% versus 15%) between “care as usual (control) groups” and the non-antibiotics groups is 78.8%. Allowing for about 10% loss to follow-up a total of 560 (4 x 140) patients will be included in the two trials.

- 1. **Data analysis**

**8.2.1 General approach**

The data-analytic approach involves data-quality management and data-analysis. Flawless data-files will be ensured by double data-entry procedures. Data-analysis proper will follow four steps: (1) univariate data checks to assess distributions and identify potential outlying values, (2) relevant bivariate analyses using tables and scatter plots, to assess crude associations between co-variables and the outcomes, and among co-variables, (3) stratified analyses and Kaplan-Meier analysis aimed at the treatment effects, (4) (Cox) regression models.

**8.2.2 Primary outcome**

Data will be analysed according to the intention to treat principle. In the two trials, the (cumulative) proportions of symptomatic UTIs at 12 months will be compared between the groups (non-inferiority part), and the time intervals to the first occurrence of faecal and urine antibiotic resistance will be compared between the two groups using standard survival time analysis techniques (resistance part).

- - 1. **Secondary outcome**

Data differences between the proportions showing adverse effects at 12 months will be analysed similarly as the primary ones, except the quality of life measures at 12 months, for which multivariable linear regression will be used. Since antibiotic resistance is a gradual phenomenon, a more refined analysis using ordinal logistic regression, will assess differences in proportions of patients at different concentrations of antibiotic resistance between the treatment groups. Other secondary analyses pertain to all microbiological outcomes at 15 months.

In both trials we will also separately analyse the patients with uncomplicated versus complicated UTIs.

**9 Economic evaluation**

Women are afflicted by urinary tract infections (UTIs) at a rate that is much higher than that for men. Although UTIs are often regarded as common or innocuous, they are associated with a considerable expense to society and nuisance for patients. This economic evaluation will be undertaken to answer the relevant decision, within two separate clinical studies, to select the most economically efficient non-antibiotic treatment versus antibiotic prophylaxis (‘care as usual’) compared in pre- en post-menopausal women with recurrent UTIs. These prospective, comparative cost studies are set up alongside the randomized clinical trials RCT-A and RCT-B.

Costs are defined from the societal perspective, regarding direct medical and non-medical and indirect non-medical costs associated with inpatient and acute outpatient management of UTI. Total resource costs are related to clinical cure, the risk of recurrent infection and evolving bacterial resistance issues and therefore reflect the economic efficiency of cranberry capsules or capsules with two strains of lactobacilli (non-antibiotic prophylaxis) compared to antibiotic prophylaxis (‘care as usual’), at the medium-term (12 months after study inclusion).

The economic evaluation will be designed as a cost-effectiveness analysis (CEA). Thereby, primary analyses will offset incremental costs (measured in Euro) against gains in the primary endpoints (number of recurrence of symptomatic UTI and time until the first occurrence of antibiotic resistance in urine or faeces) secondary analyses against quality of life years (QALYs) gained. Cost effectiveness will be calculated cost per prevented UTI. The time horizon of this cost-analysis will be limited to the study-period. The costs of treatment will be discounted against 4%. Cost reduction and better outcomes may go hand in hand, as complications associated with UTIs or treatment failures will generate additional hospital stay, patient re-evaluation, return visits, patient dissatisfaction and pharmacological reservicing costs associated with a new course of therapy.

Unit costs, if available, of used resources will be determined based on current Dutch guidelines (22). Data on resources used for in/out-patient treatment are collected from the hospital information system (with continuous registration) and financial reports. The lack of available information of medical and non-medical resource use in the patient setting and productivity loss implies more comprehensive data collection. Therefore, complementary volumes will be retrieved by self-reported questionnaires during the study period. Protocol driven costs will be excluded from the calculations.

The economic analysis will be expanded with a sensitivity-analysis to extrapolate the consequences of implementation and actual performance of using non-antibiotic treatment in (non-)complicated UTIs. In a sensitivity analysis different scenarios will be evaluated in relation to the generalisability of the results. A simulation model will be developed, based on the statistical results for subsequent analyses (1) evaluating the sensitivity for primary and secondary endpoints as well as cost parameters and (2) extrapolating to the Dutch target population and (depending on medium-term outcomes) to long-term scenarios.

**10 Ethical considerations**

**10.1 Medical Ethics Committee**

Prior to implementation the protocol and the informed consent document and any subsequent modifications will be reviewed and approved by the ethics committee responsible for oversight of the study. The study coordinator is responsible for preparation of all documents and periodic reports required by the Medical Ethics Committee.

All investigators have to ensure that his trial will be conducted in accordance with the ethical principles that have their origin in the Declaration of Helsinki, and that are consistent with Good Clinical Practice.

- 1. **Informed consent**

The consent form will describe the purpose of the study, the procedures to be followed, and the risks and benefits, and the voluntary nature of participation. Prior to consent these items will be discussed and questions from subjects will be answered. Afterwards signed informed consent will be obtained by the study coordinator. A copy of the consent form will be given to the subject, and this fact will be documented in the subject’s record.

10.3 Subject confidentiality

All laboratory specimens, evaluation forms, reports, and other records that leave the site will be identified only by SID to maintain subject confidentiality. All records will be kept away from unauthorised persons. All computer entry and networking programs will be done using SIDs only. Clinical information will not be released without written permission of the subject, except as necessary for monitoring by the medical ethics committee.

10.4 Study modification/discontinuation

The study may be modified or discontinued at any time by the Medical Ethics Committee, or specific government agencies as part of their duties to ensure that research subjects are protected.

**10.5 Benefits and risks**

Participation in this study may help participants to reduce the number of symptomatic urinary tract infections they will experience. Study participants may experience discomfort associated with collecting material for urine, faecal or vaginal cultures.

Furthermore the trial will provide information on whether non-antibiotic prophylaxis can be considered as a serious alternative for antibiotic prophylaxis (that is care as usual) in pre- and post-menopausal women with recurrent UTIs.

11 Publication policy

The NAPRUTI-study is anticipated to result in several publications regarding the effectiveness, safety and cost-effectiveness of non-antibiotic prophylaxis compared to antibiotic prophylaxis (that is care as usual) in pre- and post-menopausal women with recurrent UTIs.

The Writing Committee of the main publication(s) will consist of the study coordinator and the principal investigators. Other members of the project group will be co-authors when they have made a significant contribution to the (subject of) the article. The order of co-authors will be determined by the amount of work which is provided by each of them.

All investigators not listed as co-authors will be acknowledged and will be individually listed in an appendix (as permitted by the scientific journal).

**Reference List**

**1. Haaren van K, Visser HS, Vliet van S, Timmermans AE, Yadava R, Geerlings SE, Riet ter G, Pinxteren van B. NHG-Standaard Urineweginfecties. Huisarts en Wetenscha 199.**

**2. Foxman B. Epidemiology of urinary tract infections: incidence, morbidity, and economic costs. Dis.Mon. 2003 Feb;49(2):53-70.**

**3. Wolfhagen MJHM, Hoepelman IM. Acute urineweginfecties samengevat. In:Volksgezondheid Toekomst Verkenning. RIVM; 2003.**

**4. Ellis AK, Verma S. Quality of life in women with urinary tract infections: is benign disease a misnomer? J.Am.Board Fam.Pract. 2000 Nov;13(6):392-7.**

**5. Gezondheid op koers? In: volksgezondheid Toekomst Verkenning. RIVM; 2002.**

**6. Albert X, Huertas I, Pereiro II, Sanfelix J, Gosalbes V, Perrota C. Antibiotics for preventing recurrent urinary tract infection in non-pregnant women. Cochrane.Database.Syst.Rev. 2004;(3):CD001209.**

**7. Kunin CM. Chemoprophylaxis and suppressive therapy in the management of urinary tract infections. J.Antimicrob.Chemother. 1994 May;33 Suppl A:51-62.**

**8. Fihn SD. Clinical practice. Acute uncomplicated urinary tract infection in women. N.Engl.J.Med. 2003 Jul 17;349(3):259-66.**

**9. Gupta K. Addressing antibiotic resistance. Dis.Mon. 2003 Feb;49(2):99-110.**

**10. Murray BE, Rensimer ER, DuPont HL. Emergence of high-level trimethoprim resistance in fecal Escherichia coli during oral administration of trimethoprim or trimethoprim--sulfamethoxazole. N.Engl.J.Med. 1982 Jan 21;306(3):130-5.**

**11. Pabich WL, Fihn SD, Stamm WE, Scholes D, Boyko EJ, Gupta K. Prevalence and determinants of vaginal flora alterations in postmenopausal women. J.Infect.Dis. 2003 Oct 1;188(7):1054-8.**

**12. Reid G, Charbonneau D, Erb J, Kochanowski B, Beuerman D, Poehner R, Bruce AW. Oral use of Lactobacillus rhamnosus GR-1 and L. fermentum RC-14 significantly alters vaginal flora: randomized, placebo-controlled trial in 64 healthy women. FEMS Immunol.Med.Microbiol. 2003 Mar 20;35(2):131-4.**

**13. Raz R, Stamm WE. A controlled trial of intravaginal estriol in postmenopausal women with recurrent urinary tract infections. N.Engl.J.Med. 1993 Sep 9;329(11):753-6.**

**14. Howell AB. Cranberry proanthocyanidins and the maintenance of urinary tract health. Crit Rev.Food Sci.Nutr. 2002;42(3 Suppl):273-8.**

**15. Ahuja S, Kaack B, Roberts J. Loss of fimbrial adhesion with the addition of Vaccinum macrocarpon to the growth medium of P-fimbriated Escherichia coli. J.Urol. 1998 Feb;159(2):559-62.**

**16. Kontiokari T, Sundqvist K, Nuutinen M, Pokka T, Koskela M, Uhari M. Randomised trial of cranberry-lingonberry juice and Lactobacillus GG drink for the prevention of urinary tract infections in women. BMJ 2001 Jun 30;322(7302):1571.**

**17. Howell AB, Foxman B. Cranberry juice and adhesion of antibiotic-resistant uropathogens. Jama-Journal of the American Medical Association 2002 Jun 19;287(23):3082-3.**

**18. Foda MM, Middlebrook PF, Gatfield CT, Potvin G, Wells G, Schillinger JF. Efficacy of cranberry in prevention of urinary tract infection in a susceptible pediatric population. Can.J.Urol. 1995 Jan;2(1):98-102.**

**19. Raz R, Chazan B, Dan M. Cranberry juice and urinary tract infection. Clin.Infect.Dis. 2004 May 15;38(10):1413-9.**

**20. Terris MK, Issa MM, Tacker JR. Dietary supplementation with cranberry concentrate tablets may increase the risk of nephrolithiasis. Urology 2001 Jan;57(1):26-9.**

**21. MHRA/CSM. Interaction between warfarin and cranberry juice: new advice. Current Problems in Pharmacovigilance 2004;30:10.**

**22. Oostenbrink JB, Koopmanschap MA, Rutten FF. Standardisation of costs: the Dutch Manual for Costing in economic evaluations. Pharmacoeconomics. 2002;20(7):443-54.**

**Appendix I: List of definitions**

### Indication for prophylaxis: Patients with at least 3 or more symptomatic UTIs in the previous year before study inclusion.

### Postmenopausal women are all women, who have had their last menstruation at least one year before inclusion in the study.

### Premenopausal women are all women, who are not postmenopausal women.

### Asymptomatic bacteriuria: Presence of at least 10e3 CFU/ml of one or two micro-organisms in a culture of a clean voided urine sample isolated from a patient without symptoms of a UTI.

### Symptomatic UTI: Presence of bacteriuria (10e3 CFU/ml of one or two micro-organisms) in combination with at least one of the following symptoms: dysuria, frequency, haematuria, lower abdominal pain, flank pain or unexplained fever.

Complicated UTI: UTI in all men, pregnant women, women with structural or functional abnormalities of the urinary tract, or who are immunocompromised (e.g. having diabetes) or use immunosuppressive drugs.

Uncomplicated UTI: UTI without characteristics of a complicated UTI.
