# Supplementary material for: Cost-Effectiveness of Cranberries vs Antibiotics to Prevent Urinary Tract Infections in Premenopausal Women: A Randomized Clinical Trial
Source: PLoS One. 2014 Apr 4;9(4):e91939. doi: 10.1371/journal.pone.0091939 (PMC3976255; doi:10.1371/journal.pone.0091939)
Supplement: Table S1 — Included side effects and associated costs. (DOCX) [file pone.0091939.s003.docx]

Table S1. Included side effects and associated costs.

| **Event** | **Resource utilization** | **Costs (€, 2009)** |
| --- | --- | --- |
| Severe allergic reaction | 1 GP visit  1 A&ED visit  1 hospital admission day  0.3 mg epinephrine  1 week 2dd 1 mg clemastin | 479.25 |
| Rash or urticaria | 1 GP visit  1 week 2dd 1 mg clemastin | 28.58 |
| Nausea, vomiting, or diarrhea | 1 GP visit  2 days 3dd 10 mg metoclopramide (nausea/vomiting)  or  2 days 1dd 16 mg loperamide (diarrhea) | 35.38 |
| Constipation | 1 GP visit  1 month macrogol | 33.70 |
| Vaginal complaints | 1 GP visit  3 days 1dd 400 mg miconazol | 34.35 |
